# Impact of Different Sugar Types and Their Concentrations on Salted Duck Egg White Based Meringues

**DOI:** 10.3390/foods11091248

**Published:** 2022-04-26

**Authors:** Somwang Lekjing, Ittiporn Keawpeng, Karthikeyan Venkatachalam, Seppo Karrila

**Affiliations:** 1 Faculty of Innovative Agriculture and Fishery Establishment Project, Prince of Songkla University, Surat Thani Campus, Muang, Surat Thani 84000, Thailand; somwang.s@psu.ac.th; 2 Faculty of Agricultural Technology, Songkhla Rajabhat University, Muang, Songkhla 90000, Thailand; ittiporn.ke@skru.ac.th; 3 Faculty of Science and Industrial Technology, Prince of Songkla University, Surat Thani Campus, Muang, Surat Thani 84000, Thailand; seppo.karrila@gmail.com

**Keywords:** salted duck egg white, sugar types, meringue, functional properties, physico-chemical properties

## Abstract

Meringues were prepared with salted duck egg white and different sugars (granulated white, cane, palm, and coconut) at various concentrations (25, 50, 75, and 100%). The prepared meringues were subjected to analyses of functional and physicochemical properties and antioxidant activities. The results showed that the type and concentration of sugar influenced the meringue quality. Foam properties such as the whipping index, the index of foam durability, and specific density gradually increased with sugar concentration. On the other hand, the overrun and air phase values were stable without significant differences. The color attributes whiteness and the chroma hue of meringues decreased with the sugar concentration regardless of the sugar type. Moisture, water activity, and pH decreased, while weight loss during baking, meringue volume, and hardness gradually increased with sugar concentration. The addition of sugar increased the meringue’s weight, while its height and diameter did not change much. Protein content in the meringue decreased with increased sugar levels, while carbohydrate and ash levels increased. Antioxidant activities increased with the sugar concentration, and unrefined sugars influenced the radical scavenging activities differently from refined sugars. In general, meringue made with coconut sugar at 75% showed preferable qualities over the other sugar types or concentrations.

## 1. Introduction

Meringue is a famous confectionery product made of simple ingredients, namely whipped egg white and sugar, occasionally with added acidic ingredients. The foaming properties of the egg white are the basis for the characteristic texture of meringue. The whipping breaks the hydrogen bonds of the albumin protein in the egg white and creates a white thin-filmed foam with entrapped air, while added sugar and acidic substances stabilize and strengthen the foam [1]. While the word ‘meringue’ is not commonly used in Thailand, the product is very famous and widely used and is called ‘egg white sweet’ or ‘crisp egg white sweet.’ The main ingredient, egg white, serves as the prime source of poultry proteins [2], and the secondary ingredient, sugar, plays a significant role in the physicochemical and sensory properties. The sugar acts as a stabilizer by binding with the water and giving a stable shape to the meringue. The addition of sugar is the critical point in the production of meringue, as it can reduce the foam formation in the beginning and has to be gradually added only after the foaming has begun [3]. The sugar also enables the meringue to stretch and have a high content of air bubbles that can make the meringue light and airy. Typically, refined white granulated sugar is used; however, it is a simple carbohydrate with no beneficial health effects, although it does improve consumer perception.

Thailand is among the major producers of unrefined sugars, primarily cane, palm, and coconut sugars, widely marketed in Thailand and used to make various Thai desserts. Recent studies have found that the addition of unrefined sugars to food product processing has contributed to many nutritional benefits compared to refined granulated sugars [4]. Furthermore, these unrefined sugars are well known for their antioxidant activities, as they contain an abundance of polyphenolics, particularly gallic acid, protecatechuic acid, caffeic acid, p-coumaric acid, etc. [5]. In addition to sugar, the baking process also contributes to antioxidant activities in meringue, as Charoen et al. [6] found that the baking process increased the formation of Schiff base products in meringue and thus established the metal chelating ability of meringues. Furthermore, the egg white proteins including ovotransferrin, ovomucoid, lysozyme, and ovomucin contributed as strong antioxidants in food products [7]. Awatsuhara et al. [8] reported that when egg white proteins are combined with natural polyphenols, they can increase the antioxidant activities upon thermal processing. Antioxidants do not contribute to the taste profile of the food products; however, they are well known for their protection against various chronic diseases generated by oxidative stress [7].

Thailand is famous for salted duck eggs, and it is one of the primary producers of these eggs in South-East Asia. Salted duck eggs serve as an essential condiment mainly in Chinese and Northeastern Thai cuisines [9]. The duck eggs are usually subjected to extensive salting to obtain a harder, glossier, and tastier egg yolk, while the egg white, as the primary receiver of the salt, becomes too salty. The salted egg whites are then too unpleasant for consumption, but this byproduct holds a considerable amount of protein, and discarding it is an economic loss as well as an environmental problem [10]. The salt content in the salted duck egg white varies between 4 and 8%, and it depends on the salting method, such as immersion in brine and/or covering with salty mud coating [11]. The eggs coated with a salt-infused mud coating usually reach around 4–6% salt infusion in the egg white. Various studies have been conducted to reduce the waste of salted duck egg whites, testing treatments such as desalination, hydrolyzation, filtration, etc. [12]. These techniques are promising for laboratory-based work but are currently not practically applicable to general consumers and producers. Developing food that utilizes this salted duck egg as an ingredient would be an excellent approach to waste management. Therefore, the present study aimed to develop a meringue utilizing salted duck egg whites as the key ingredient and test it with various native sugars at different concentrations to investigate the impacts on suitability, functional, and physicochemical properties.

## 2. Materials and Methods

### 2.1. Collection of Raw Materials

The salted duck eggs were purchased from local producers in Chaiya district, Surat Thani province. The acquired eggs (20 days salt-cured, 2.8% NaCl) were thoroughly washed and cracked open to separate the egg white (EW) to make meringue. The other ingredients, namely alternative types of sugar (white, cane, coconut, or palm) and stabilizers such as cream of tartar, were purchased from a local supermarket in Surat Thani province.

### 2.2. Development of Meringue

The meringue was prepared with various proportions of the ingredients relative to EW (100%): Sugar (25, 50, 75, or 100%) and stabilizer (0.01%, cream of tartar). The EW was whipped using an electric kitchen mixer (Cuzimate, RBSFOODMIXERPRO, Cuizimate, Thailand) with a 4.5 L stationary bowl and rotating beaters. The speed was initially kept low (2000 rpm) for 5 min then increased (4000 rpm), and whipping was continued for 10 min more. The sugar and stabilizer were gradually added, both at the same time, to obtain a homogeneous mix. The whipping was then measured for foam properties, after which it was transferred to a pastry bag equipped with a nozzle (3 cm). A small cone-shaped meringue was made with the pastry bag on a parchment-lined baking sheet set on the baking tray, and after that, the meringues were placed in a double-decker infrared oven set at 163 °C for 25 min of baking [13]. The baked meringues were cooled to room temperature and then subjected to quality determinations. The optimum composition of ingredients for making meringues was identified based on functional and physico-chemical parameters.

### 2.3. Analyses

#### 2.3.1. Foam Characteristics

The foam characteristics—the index of whipping (IW), index of foam durability (ID), specific density (SD), overrun (OR), and air phase (AP)—were measured using the methods of Bovskova and Mikova [13] with slight modifications.

#### 2.3.2. Hardness

The texture in terms of the hardness of the baked meringue was measured using a texture analyzer (Model no. CT3, Brookfield Texture Analyzer, Middleboro, Massachusetts, USA) in compression mode with a sharp-blade cutting probe. Pre-test, test, and post-test speeds of 1.5, 2, and 10 mm/s were applied to determine the hardness. The hardness was measured for 15 replicate meringues, and the force in N is reported as the hardness measure [13].

#### 2.3.3. Weight Loss during Baking

Water loss (WL) during baking was determined by weighing the meringue after 24 h of baking. The following equation [14] was used:

**WL (%) = [(B − C)/W] × 100**

where WL is the weight loss during baking, B is the weight (in grams) of the meringue before baking, C is the weight (in grams) of the meringue after baking, and W is the initial water content (in grams). To calculate the W, the initial water content in each formulation ingredient was taken into account.

#### 2.3.4. Meringue Volume

The meringue volume was immediately determined after baking by the rapeseed displacement method, i.e., filling a glass container of known volume uniformly with rapeseeds and tapping and smoothing the surface with a ruler. The constant weight reached was recorded for each consecutive measurement. The volume of the sample was calculated as follows:

**Wseed = Wtotal – Wsample − Wcontainer**

**Vseed = Wseed/Pseed**

**Vsample = Vcontainer − Vseed**

where W is the weight (kg), seed is the rapeseed, sample is the meringue, V is the volume (m^3^), and P is the density (kg/m^3^).

#### 2.3.5. Measurement of Diameter, Height, and Weight

The diameter, height, and weight measurements of the meringue followed the method of Zoulias et al. [15] with slight changes. The diameter and height of the meringue were measured with a Vernier caliper at three different places, and the average was taken as the final measured quantity. The weight of the meringue was measured using a weighing balance.

#### 2.3.6. Measurement of Moisture, Water Activity, and pH

The moisture content of the meringue was measured using an infrared moisture analyzer (MA160, Sartorius, Göttingen, Germany). The water activity was measured at 25 °C with a dew-point water activity analyzer (Series 4TEV, Aqua Lab, Pullman, WA, USA). Ten grams of the sample was homogenized with 100 mL of distilled water, and pH was measured using a handheld digital pH meter (Clean, pH30, New Taipei City, Taiwan)

#### 2.3.7. Proximate Composition and Energy Value

The proximate composition in terms of protein, ash, and carbohydrate contents in the meringue was calculated. Protein was analyzed by a protein analyzer (CN-Analyzer, Leco, CHN628, St. Joseph, MI, USA) following the method of AOAC [12]. The carbohydrate contents of meringue samples were determined according to the method of AOAC [16]. Small pieces of meringue (2 g) were put in a crucible that was then placed in a muffle furnace for ash determination. The samples were subjected to heating at 550 °C for 3 h. The weight of the matter remaining after incineration was calculated as follows:

**% Ash = ((b − a)/w) × 100**

where a is the weight of the crucible, b is the weight after incineration of the crucible with the sample, and w is the dry weight of the sample.

The energy value of the end-product was measured using an Energy analyzer (Bomb Calorimeter, AC500, Leco, St. Joseph, MI, USA). The sample was crushed into a fine powder and placed in a pellet analyzer into the bomb calorimeter, and the energy value was recorded.

#### 2.3.8. Pictorial Reference and Color Profile Analysis

Digital photographs of meringue samples after preparation were captured for pictorial reference using a handheld digital camera (Compact camera, ZV, Sony, Minato, Tokyo, Japan). The surface color of the meringue was determined using the CIE color coordinates L* (lightness), a* (Chroma), and b* (Hue) using a Hunter Colorimeter (Hunter Assoc., Reston, VA, USA) [16].

#### 2.3.9. Determination of Antioxidant Activities

Prior to determination, 5 g of meringue samples were collected and coarsely ground into a powder and suspended in a test tube containing 10 mL of ethanol (95%) followed by vigorous vortexing for 3 min. After that, the samples were collected and centrifuged at 10,000× *g* for 20 min (4 °C). Then, the supernatant was carefully collected and stored in a cold and dark place for subsequent analysis. For the DPPH (2,2-diphenyl-1-picrylhydrazyl) radical determinations [17], a 100 μL aliquot was thoroughly mixed with 3.9 mL of 60 μmol/L DPPH, and after that, the reaction mixture contained in test tubes were covered and placed in the dark at room temperature for 30 min. Following that, the reaction mixture was used to measure the absorbance reading at 515 nm using a spectrophotometer. The obtained results were expressed as a percentage of the ability to reduce the DPPH radicals. For ABTS (2,2′-azino-di-3-ethylbenzthi¬azoline sulfonic acid) radical scavenging activity [18], a 100 μL aliquot was mixed with100 μL of the ABTS reagent in a test tube and then placed in a 96-well microplate followed by incubation for 6 min at room temperature in dark conditions. After incubation, the samples were measured at 734 nm using a microplate reader. The obtained results were expressed as a percentage of the ability to reduce the ABTS radicals. For hydroxyl radical scavenging activity [19], a 1 mL aliquot was added to a test tube followed by the addition of 0.2 mL of the reaction mixture containing 0.2 mL of 10 mmol ferrous ammonium sulfate, 0.2 mL of 10 mmol EDTA, and 0.6 mL of 0.1 mol phosphate buffer (pH 7.4). After that, 0.2 mL of hydrogen peroxide was added to the test tube and the volume was made up to 2 mL using 0.1 mmol phosphate buffer (pH 7.4). Then, the reaction mixture was thoroughly mixed and incubated at room temperature for 4 h, and after incubation, 1 mL of 1% thiobarbituric acid and 1 mL of 2.8% trichloroacetic acid were added to the test tube and mixed well. After that, the whole reaction mixture was kept in a boiling water bath for 10 min followed by cooling in an ice bath, and then used to measure the absorbance at 520 nm using a spectrophotometer. The obtained results were expressed as a percentage of the ability to reduce the hydroxyl radical. For ferrous ion chelating activity [20], a 200 µL aliquot was added to a test tube followed by 0.8 mL of 95% ethanol that was mixed with 5 mM ferrozine and 2 mM FeCl_2._ Following that, the reaction mixture was mixed well and then allowed to incubate for 10 min at room temperature. Then, the mixture was measured at 562 nm against the control. The control solution contained 0.2 µL of EDTA prepared in 95% ethanol instead of the sample in the reaction mixture. The obtained results were expressed as a percentage of the ability to reduce the ferrous ion.

### 2.4. Statistical Analyses

All data presented in this research are reported as mean values with standard deviations from triplicate experiments. A completed randomized design was applied in the experiments. The significant differences (*p* < 0.05) between the means of the analysis were explored and compared using the one-way analysis of variance (ANOVA) and Duncan’s multiple range test. The statistical analyses were performed using SPSS (SPSS Inc., Chicago, IL, USA) for Windows.

## 3. Results and Discussion

### 3.1. Foam Characteristics

**Figure 1:**
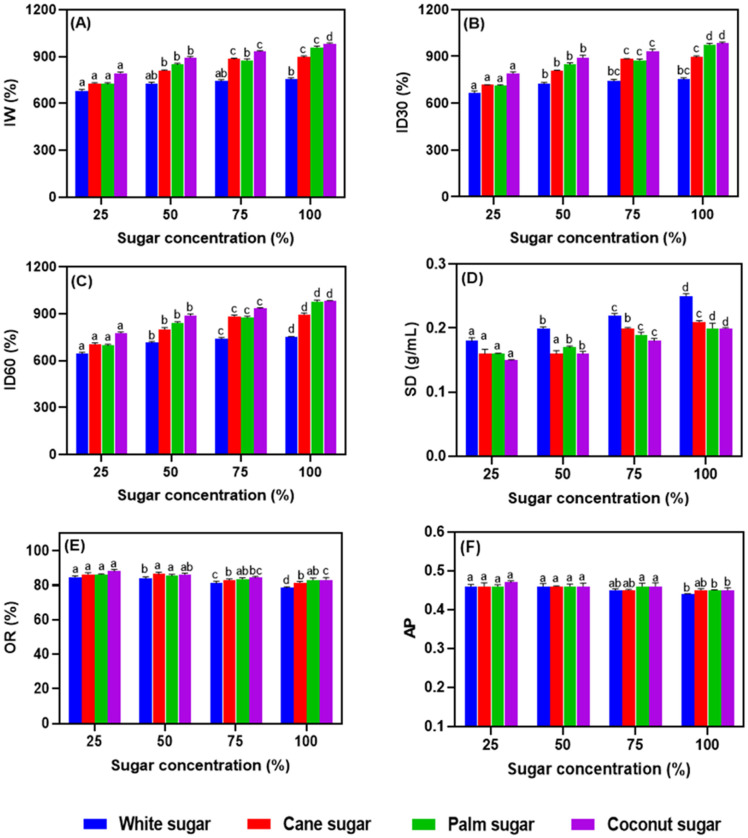
Functional properties (**A**–**F**) of salted duck egg white foam prepared with alternative types of sugar at various levels in the formulation. Note: IW stands for index of whipping, ID stands for index of foam durability, SD stands for specific density, OR stands for overrun, and AP stands for air phase. Different alphabets above the bars indicate significant differences among various conditions.

The foam characteristics of the salted duck egg white incorporated with alternative sugars at various concentrations are shown in Figure 1A–F. The foam characteristics such as the index of whipping (IW), index of foam durability (ID), specific density (SD), overrun (OR), and air phase (AP) were affected by the sugar type and its concentration. Egg white protein albumen has excellent foaming properties [21]. However, even minute levels of egg yolk can negatively influence the foaming ability of egg albumen. The IW was notably affected by coconut sugar, followed in rank order by palm, cane, and white sugars. A high concentration of coconut sugar increased the IW over those with the other sugars. The ID of the foam was also affected by the coconut sugar at high concentrations. There was no significant difference in the ID values at 30 and 60 min of meringue samples (*p* > 0.05). In contrast, the foam’s SD was highest with refined white sugar, followed in rank order by cane, palm, and coconut sugars (*p* < 0.05). The results also show that SD increased with sugar concentration. On the other hand, the OR and AP of the foam had no significant differences across the type of sugar or its concentration (*p* > 0.05). Sugar plays a significant role in influencing the stability and rheological properties of the egg white foam. The incorporation of sugar, especially dextrose, lactose, or maltose, initially delays the foaming [22]. Sadahira et al. [23] found that manipulation of the pH could stabilize and/or destabilize egg foam, and a low pH (3 to 4.5) showed a positive impact on stability. While a low pH improved foam stability, it decreased foaming capacity, while both foaming capacity and foaming stability became feeble at higher pH. An increased sugar concentration could remedy this issue and improve the egg foam’s rheological properties. The present study is in agreement with these phenomena, as increasing the sugar concentration in the meringues gradually improved the functional properties. This might be due to the slight decrease in pH in the meringues when the sugar concentration was increased. Lomakina and Mikova [1] reported that the foaming capacities of egg white improved when the pH ranged from acidic to neutral. The study also reported that the addition of sugar could delay foam formation. Hartel et al. [24] stated that the addition of sugar at the end of foam production was not able to adversely affect the foaming capacity of egg white. Furthermore, the source of egg white also plays a major role in the foaming properties. Ho et al. [25] studied the foaming characteristic of unsalted hen egg whites and the results were vastly different, especially for the foam’s overrun and air phase, and the density was slightly lower in hen egg white foams as compared to the present study.

### 3.2. Pictorial Reference and Color Profile

**Figure 2:**
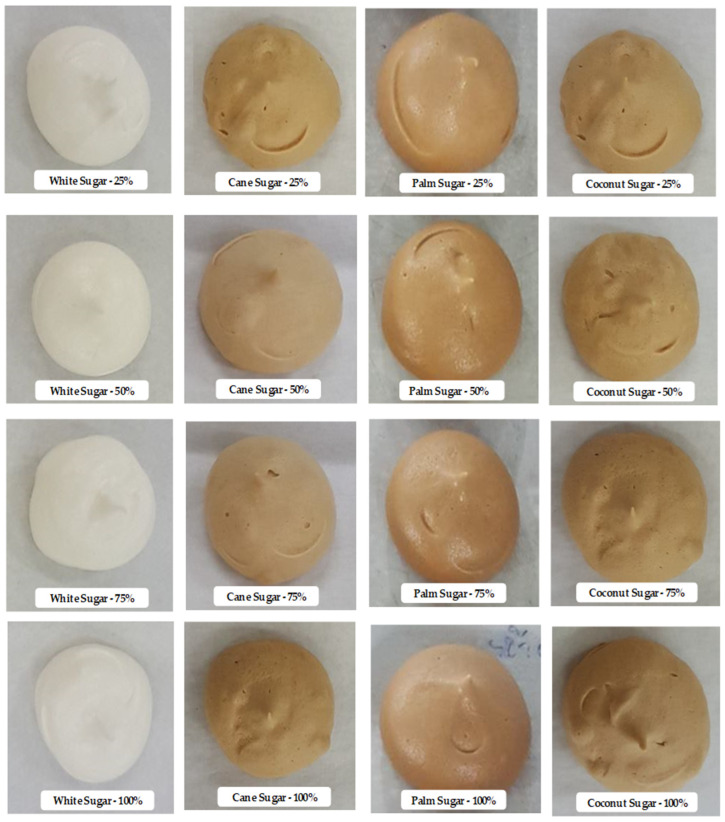
Pictorial reference of salted duck egg white meringues prepared with alternative types of sugar at various levels in the formulation.

**Figure 3:**
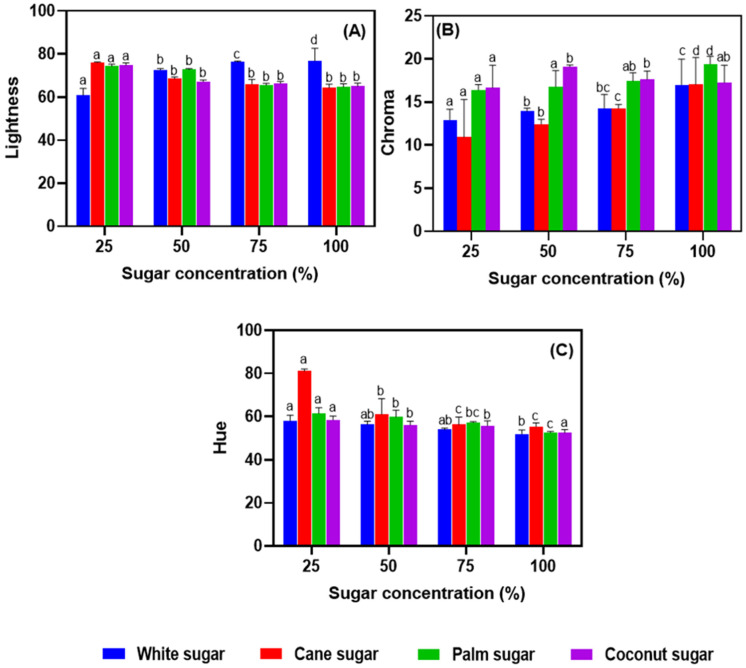
Color profile (lightness (**A**), chroma (**B**), and hue (**C**)) of salted duck egg white meringues prepared with alternative types of sugar at various levels in the formulation. Different alphabets above the bars indicate significant differences among various conditions.

The pictorial references of the meringues prepared with different types of sugar at different concentrations are shown in Figure 2. The appearance of the meringue samples was significantly affected by the sugars. The increased concentration of sugar in the meringue greatly influenced the appearance of the meringues. Similarly, the color profiles (lightness (L*), chroma (a*), and hue (b*) of the meringues prepared with the different types of sugar at different concentrations were also greatly affected (Figure 3A–C). The meringues made with high concentrations of white sugar showed the highest lightness values among the different sugars. On the other hand, cane, palm, and coconut sugars tended to decrease in lightness with an increasing concentration. However, there were no significant differences in lightness at the highest concentration used (100% relative to egg white) (*p* < 0.05), while the lightness was lower than with 25% sugar. Hue, which represents the yellowness, was high with cane sugar at the 25% level; in contrast, the same sugar resulted in better hue values when the sugar concentrations increased gradually to 100%. The chroma of meringues increased with the sugar concentration regardless of the sugar type. Meringue made with palm or coconut sugar had a higher chroma value than when made with white or cane sugar (*p* < 0.05). The Maillard reaction during the baking of the meringue plays a significant role in the color profile, as it produces brown pigmented byproducts, particularly melanoidins [26]. In addition, the color of the meringue is also affected by the egg type. Yuceer and Asik [27] observed the color profiles of hen egg white meringues and found that the meringue made of granulated white sugar was slightly brighter than the present study, which indicates that salted duck egg white has a naturally slightly lower color profile. Charoen et al. [6] reported that redness increases with storage time owing to the Maillard reaction. It should also be noted that protein-rich products are generally prone to color changes. Damodaran [28] reported that the Maillard reaction rate is affected by the nature of the reducing sugar used. Different sugars exhibited different reaction rates, and simple sugar with high glucose levels showed a higher reaction rate than other monosaccharides or polysaccharides. Phaichamnan et al. [29] reported that differences in the reducing sugar might be caused by contamination with microorganisms that convert sucrose into invert sugars (mainly glucose and fructose) and further to organic acids and alcohols. Kongkeaw et al. [30] observed a high level of reducing sugar in coconut sugar, followed by palm, cane, and white sugars. This is in accordance with the present study, namely that a higher level of reduction in the sugar type affected the interaction with the egg white and influenced the meringue’s color profile. Curi et al. [31] reported that sugar type significantly influenced the physicochemical properties, particularly the color of physalis jelly.

### 3.3. Weight, Height, and Diameter of Meringues

**Figure 4:**
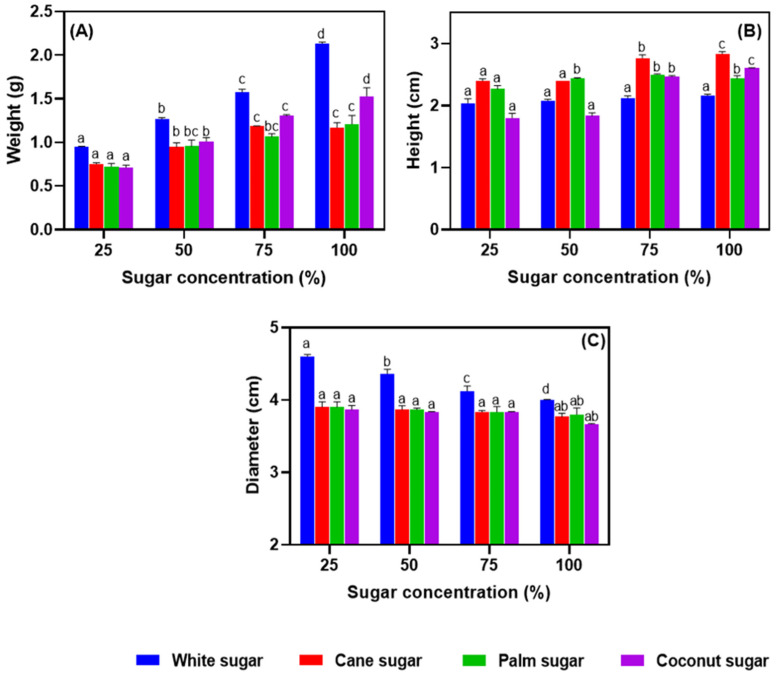
Weight (**A**), height (**B**), and diameter (**C**) of salted duck egg white meringues prepared with alternative types of sugar at various levels in the formulation. Different alphabets above the bars indicate significant differences among various conditions.

The weight, height, and diameter of meringues made with different sugars and varied formulations are shown in Figure 4A–C. Time and temperature are the two significant factors affecting the physical parameters of baked food materials during processing and storage [32]. The weight of the samples gradually increased as the sugar concentration increased (*p* > 0.05). This might be due to the hygroscopic nature of sugars that plausibly absorbed moisture from the ambient air. White sugar gave the heaviest meringue, followed by coconut sugar. However, no differences between cane, palm, and coconut sugars were present at the lowest sugar concentration. On the other hand, a significant difference in the height of the meringues was found by sugar type (*p* < 0.05). Nonetheless, the differences in height were marginal, even though a higher sugar content resulted in a larger height of meringue. There was no difference between the 25 and 50% sugar concentrations. The diameter of meringue tended to decrease as the white sugar concentration was increased, but there was no significant difference in the diameter of meringues made with sugar concentrations of 25–75%, irrespective of sugar type.

### 3.4. Weight Loss during Baking, Meringue Volume, Hardness, Moisture Content, Water Activity, and pH

**Figure 5:**
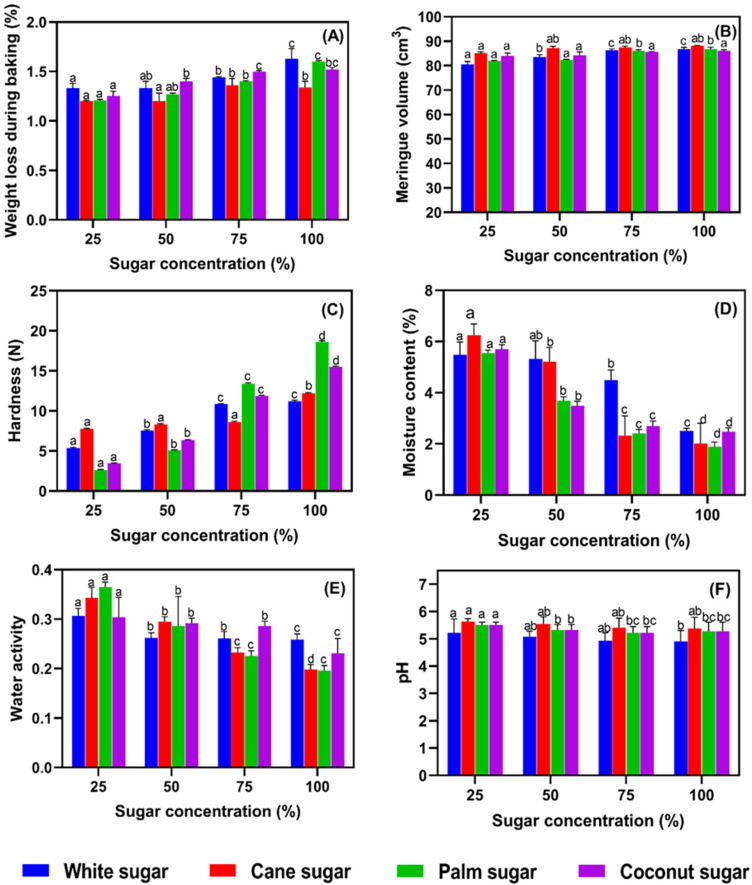
Weight loss during baking (**A**), meringue volume (**B**), hardness (**C**), moisture content (**D**), water activity (**E**), pH (**F**) of salted duck egg white meringues prepared with alternative types of sugar at various levels in the formulation. Different alphabets above the bars indicate significant differences among various conditions.

The weight loss during the baking of meringues made with alternative sugar types and various concentrations is shown in Figure 5A, and the weight loss during baking varied considerably, being higher with white and palm sugars than with others (*p* < 0.05). The results indicate that increasing the sugar concentration gradually increased the weight loss. The meringue volume slightly increased with sugar concentration (*p* < 0.05) (Figure 5B), but the differences were small and mostly non-significant. The cane sugar gave the meringue a larger volume than the other sugar types. The meringue volume is also affected by the mixer, its rotation speed, the ingredients, and the batter temperature. Furthermore, meringues with the largest volume tend to show the lowest hardness [33]. The hardness of meringue samples differed by the type and concentration of sugar used (Figure 5C). The addition of palm sugar increased the hardness of the meringue, while white sugar decreased the hardness (*p* < 0.05). However, at a low level of incorporation (25%), white sugar did not affect the hardness, while in contrast, the same concentration of cane sugar resulted in a harder meringue. The hardness of meringue largely depends on the type and composition of sugar, and sugar with mineral content gives a harder texture [31]. The moisture content and water activity (aw) of meringue samples gradually decreased with the increasing level of sugar in the meringue recipe (Figure 5D,E). Water activity has profound effects on the rheological properties and shelf life of meringues [34]. Water activity is directly related to hygroscopic moisture accumulation from ambient air and generally increases during storage [35]. In general, white sugar produced meringues with the highest moisture content among the sugar types tested. Sugar content adversely affected the moisture content and water activity of the meringues. The pH of samples was slightly elevated when using cane, palm, or coconut sugar in meringue preparation (Figure 5F), as the white-sugar-based meringues showed the lowest pH, regardless of sugar concentration (*p* < 0.05). The water activity level in the salted duck egg white meringues, particularly in the traditional refined white sugar meringues in the present study, did not differ much from the hen egg white meringues studied by Yuceer and Asik [27]. Kim et al. [36] stated that the pH of meringue is neutral in general. However, the meringue prepared from salted duck egg white showed slightly lower, mildly acidic pH.

### 3.5. Proximate Composition of Meringues

**Figure 6:**
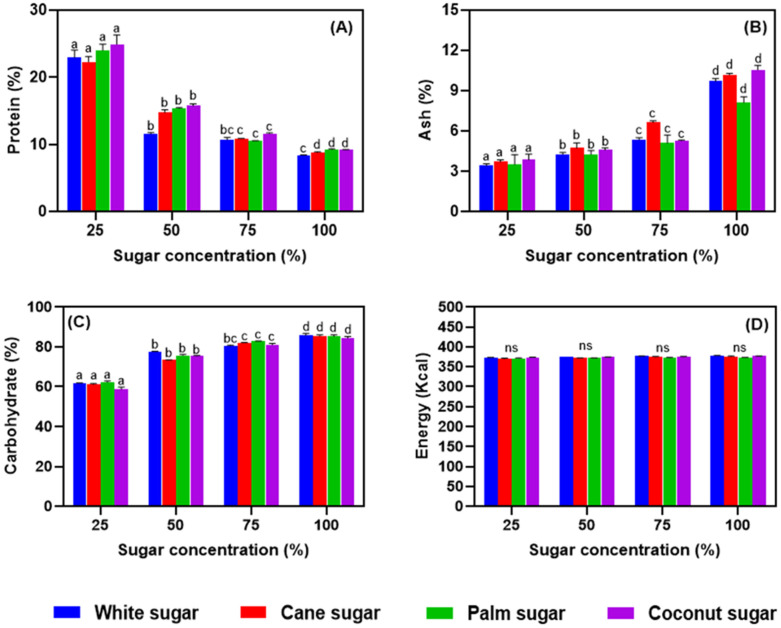
Proximate composition (**A**–**D**) of salted duck egg white meringues prepared with alternative types of sugar at various levels in the formulation. Different alphabets above the bars indicate significant differences among various conditions.

The proximate compositions and energy values of meringues are summarized in Figure 6A–D. The different sugars at various concentrations substantially affected the proximate composition of the meringues (*p* < 0.05). Moreover, the differences in the proximate composition of the meringue might have affected the proximate composition of raw materials used. The results show that protein content in the meringues did not vary by type of sugar. However, the sugar concentration adversely influenced the protein content. Kaewmanee et al. [37] reported that the salted duck egg white protein content was 10.5%, and in our present study, we found that salted duck egg white meringue with a lower sugar concentration had a higher level of protein content. Dills Jr [38] reported that an increased concentration of sugar would adversely affect the protein content by inducing the production of Maillard products and thus resulting in the loss of amino acid residues and decreased protein digestibility. Several reports found that refined granulated white sugar contains no protein, whereas cane (0.26%), palm (0.25%), and coconut sugars (0.17%) contain fractional levels of proteins [39,40]. The addition of sugar at a higher level decreased the protein content and increased the carbohydrate and ash contents regardless of sugar type. The total carbohydrate level was similar to white, cane, and palm sugars, while the meringue made with coconut sugar showed a slightly lower level of total carbohydrates. Asghar et al. [40] reported in their study that coconut sugar contained the lowest carbohydrate content as compared with palm, cane, and white sugar. The ash content increased with the concentration of sugar, regardless of sugar type. However, the lowest ash content was found for meringues prepared with white and palm sugars (*p* < 0.05). Curi et al. [31] reported that unrefined sugar contains minerals such as Fe, Zn, Ca, and K, and vitamins B1, B2, B3, and B6, along with short-chain fatty acids, polyphenols, antioxidants, etc., that have enormous health benefits. Furthermore, the increased level of these sugars in the meringue composition increased the ash content in the samples. de Bruijn and Bout [41] reported that white sugar contains many cations and anions and these interact with the salted duck egg white and could increase the ash contents. Asghar et al. [40] observed that among the different sugar types, coconut sugar retained a higher ash content, followed by palm and cane sugars. An insignificant increment in the energy level of meringues was observed with the increased sugar concentration. In general, white and coconut sugars gave the meringues slightly higher energy contents than the other sugar types.

### 3.6. Antioxidant Activities of Meringues

**Figure 7:**
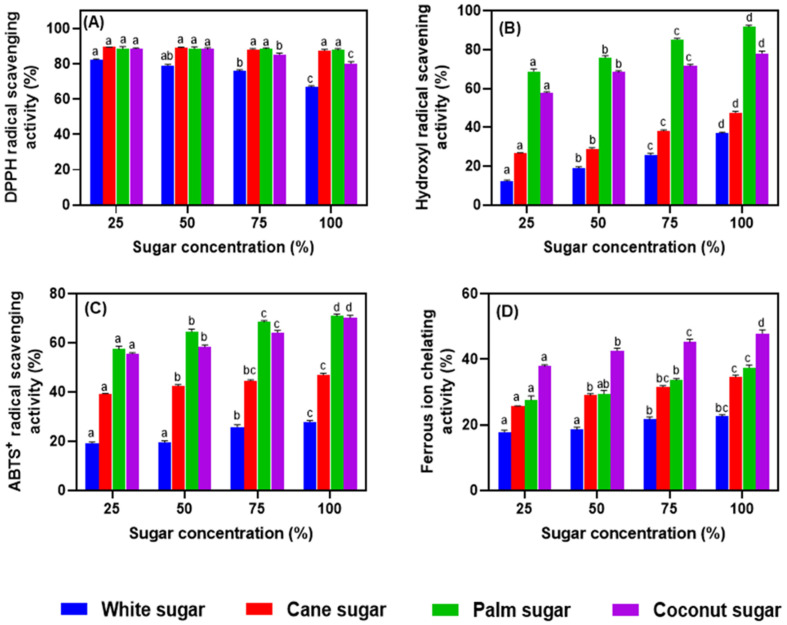
Radical scavenging capacity (**A**–**D**) of salted duck egg white meringues prepared with alternative types of sugar at various levels in the formulation. Note: DPPH stands for 2,2-diphenyl-1-picrylhydrazyl, and ABTS+ stands for 2,2′-azino-bis (3-ethylbenzthiazoline-6-sulphonic acid). Different alphabets above the bars indicate significant differences among various conditions.

The antioxidant activities of meringues made with different sugars at various levels are shown in Figure 7A–D. Raw material choices could influence the radical scavenging activities of food. The different sugars showed different radical scavenging activities, and the measurement of antioxidant activities can be of great significance. Unrefined sugars naturally exhibit radical scavenging activities. Kongkaew et al. [30] studied the antioxidant potentials of various sugar types and found that cane and palm sugars exhibit excellent radical scavenging activities. In this study, the meringues exhibited significant radical scavenging potency. The DPPH radical scavenging activity of meringues is shown in Figure 6A. White sugar meringues showed the lowest scavenging potency among the cases tested. In contrast, cane and palm sugars produced meringues with higher DPPH radical scavenging activities (*p* < 0.05). On the other hand, the coconut-sugar-based meringues showed DPPH radical scavenging activity that slightly decreased with sugar levels (75 and 100%). The hydroxyl radical scavenging activity of the meringues was higher in the samples that were prepared using palm and coconut sugars and lower with white and cane sugars (*p* < 0.05) (Figure 6B). The palm sugar produced meringues with the highest hydroxyl radical scavenging activity amongst the types of sugar tested. A similar pattern was also observed for the ABTS^+^ radical scavenging activities of the meringues. Meringues made with palm and coconut sugars exhibited better ABTS^+^ radical scavenging activities than those with white and cane sugars (Figure 6C). Meringues have high antioxidant activities owing to melanoidin and D-ketohexose contents according to Charoen et al. [6]. In general, the ferrous ion chelating activity of meringues was slightly lower than the other radical scavenging activities (Figure 6D). Meringues made with coconut sugar showed the highest ferrous ion chelating activity among the cases tested. White sugar meringues exhibited the lowest ferrous ion chelating activity (*p* < 0.05). Chaiyasut et al. [42] studied the antioxidant activities of various honey and sugars and reported that the polyphenolic content was mainly responsible for ferrous ion chelating activity. However, sugars have low phenolic contents and correspondingly low ferrous ion chelating activities. Kongkaew et al. [30] found higher phenolic contents in cane, coconut, and palm sugars than in refined white sugar. The antioxidant activity of meringue largely depends on its total phenolic content [36]. This is in accordance with the present study, in which higher antioxidant activities were found for cane, palm, and coconut sugars than for white sugar. The antioxidant activity of meringues increased with sugar level, irrespective of the sugar type.

## 4. Conclusions

This study tested and explored whether salted duck egg white is an excellent candidate for producing meringues. This study had the dual motivation of not wasting valuable duck egg white protein, a byproduct of making salted eggs, and the reduction of environmental pollution from the disposal of salted egg whites. The choice of sugar type and its level influenced the quality attributes of meringue to a great extent. It was demonstrated that unrefined sugars positively influenced various properties of meringues when compared to refined white sugar. Overall, this study demonstrates that the incorporation of coconut sugar, particularly at a 75% concentration, provided a significantly better quality as well as better functional properties compared with other sugars at the levels tested. However, a technical investigation of the sensory characteristics of these meringue samples will be conducted in further investigations to explore consumer preferability.

## Data Availability

Not applicable.
